# Clinical study of PLA2R epitope spreading for predicting proteinuria remission in primary membranous nephropathy

**DOI:** 10.3389/fimmu.2025.1685738

**Published:** 2025-10-22

**Authors:** Xueyang Cheng, Meiyi Zhou, Caimei Chen, Jing Xue, Bin Liu, Zhijian Zhang, Xiran Zhang, Leting Zhou, Ting Cai, Biao Huang, Yi Zhang, Liang Wang, Xiaobin Liu

**Affiliations:** ^1^ Department of Nephrology, The Affiliated Wuxi People’s Hospital of Nanjing Medical University, Wuxi People’s Hospital, Wuxi Medical Center, Nanjing Medical University, Wuxi, China; ^2^ College of Life Sciences and Medicine, Zhejiang Sci-Tech University, Hangzhou, China; ^3^ National Health Commission (NHC) Key Laboratory of Nuclear Medicine, Jiangsu Key Laboratory of Molecular Nuclear Medicine, Jiangsu Institute of Nuclear Medicine, Wuxi, China

**Keywords:** primary membranous nephropathy, PLA2R, epitope spreading, rituximab, anti-rituximab antibody

## Abstract

**Introduction:**

M-type phospholipase A2 receptor (PLA2R) is the predominant autoantigen in primary membranous nephropathy (PMN), accounting for approximately 70%–80% of cases. Circulating anti-PLA2R IgG is a widely used biomarker to monitor disease activity and treatment. In recent years, antibodies targeting specific PLA2R domains and epitope spreading of PLA2R have been identified and suggested to be correlated with disease severity and resistance to treatment. However, its clinical relevance remains controversial. This study aimed to evaluate whether epitope spreading offers superior prognostic value compared to total anti-PLA2R IgG levels in patients with PMN.

**Methods:**

This retrospective study enrolled 74 patients with biopsy-proven PMN who underwent at least 6 months of follow-up. Clinical data and serum samples were collected at baseline (M0), 6 months (M6), and 12 months (M12). PLA2R-IgG, domain-specific antibodies (CysR-, CTLD1-, and CTLD7/8-IgG/IgG4), and anti-rituximab antibodies (ARAs) were measured using time-resolved fluorescence immunoassay. Logistic regression and receiver operating characteristic curve analyses were used to assess prognostic factors and model performance. The patients were divided into cyclophosphamide (CTX) and rituximab (RTX) treatment groups.

**Results:**

There were no significant differences in remission rates between the groups at M6 (CTX: 37.9% vs. RTX: 60.0%, *P* = 0.875) or M12 (61.5% vs. 75.6%, *P* = 0.220). However, the RTX group showed faster antibody clearance at M6 and a significantly higher immunological remission rate at M12 (96.2% vs. 65.6%, *P* = 0.017). In the RTX group, epitope spreading significantly decreased at M6 (*P* = 0.004), and four patients (22.2%) with no clinical remission were ARA-positive. Multivariate logistic regression analysis identified epitope spreading as an independent risk factor for non-remission at M6 (*P* = 0.031; AUC = 0.932). All four ARA-positive patients achieved partial or complete remission within 3–9 months after switching to obinutuzumab.

**Discussion:**

Compared with CTX, RTX induced a higher rate of immunologic remission at M12. Epitope spreading of PLA2R was identified as an independent risk factor for clinical remission after 6 months of treatment with RTX.

## 1 Introduction

Membranous nephropathy (MN) is classified as either primary membranous nephropathy (PMN) or secondary membranous nephropathy according to the etiological classification and generally manifests as significant proteinuria (>3.5 g/24 h), hypoalbuminemia (<30 g/L), edema, hyperlipidemia, and other metabolic disorders (1, 2). PMN is an autoimmune renal glomerular disease mediated by the autoantigen antibody complex, among which phospholipase A2 receptor (PLA2R)-associated PMN accounts for 70%–80% (3).

According to the KDIGO guidelines, serum PLA2R-IgG is recommended as an efficient biomarker for risk stratification and efficacy determination in PMN. However, there are still some open questions, such as understanding the mechanisms of epitope spreading and immunodominance and determining whether the analysis of epitope reactivity has a greater predictive value than that of the PLA2R-antibody level (4). To date, a range of domain-specific epitopes of the PLA2R antigen have been identified through the discovery of specific antibodies for the PLA2R-CysR, PLA2R-CTLD1, and PLA2R-CTLD7/-CTLD8 domains. In 2016, Polski et al. demonstrated that both anti-PLA2R antibodies and epitope spreading could independently predict poor renal prognosis and serve as prognostic tools to monitor disease severity (5). However, different perspectives exist regarding the clinical significance of epitope spreading. In 2020, Reinhard et al. observed that clinical outcomes were only related to total anti-PLA2R antibody levels at the time of diagnosis but not to specific domain antibodies (6). Our previous study revealed that PLA2R-CTLD1-IgG4 might be a more effective biomarker to predict proteinuria remission at the 6th month of treatment in PMN compared with total PLA2R-IgG (7). Therefore, more studies are needed to confirm the association of epitope spreading and domain-specific antibodies with clinical remission and prognosis.

In addition, rituximab (RTX), a CD20 monoclonal antibody that eliminates autoantibodies by depleting B cells, has been recommended for patients with moderate- and high-risk PMN (4). Various studies on RTX used in PMN have also confirmed its efficacy, achieving clinical remission in 60%–80% of PMN patients (8). However, 20%–40% of PMN patients still develop resistance during RTX treatment, which may be related to the production of anti-rituximab antibodies (ARAs) (8). In 2020, Boyer-Suavet et al. enrolled 44 MN patients who were treated with RTX. Anti-rituximab antibodies were detected in 10 patients. Moreover, the rate of relapse was significantly higher in patients with ARAs (9). Changes in treatment regimens may be required for these patients. Studies on obinutuzumab, a humanized monoclonal antibody, in RTX treatment failure patients are ongoing progressively (10).

In this study, we aimed to elucidate whether epitope spreading offers superior clinical predictive value compared to PLA2R antibody levels, which will help facilitate the development of more accurate clinical prediction models in PMN.

## 2 Materials and methods

### 2.1 Patients and samples

In this retrospective study, the PMN cohort of 74 patients was followed up for at least 6 months. All patients were confirmed by renal biopsy during hospitalization in The Affiliated Wuxi People’s Hospital of Nanjing Medical University from January 2020 to December 2023 and positive for PLA2R antibody. Serum specimens were obtained at baseline (M0) and 6 (M6) and 12 months (M12) of treatment, which were processed through standardized centrifugation (3,000 rpm for 5 min), cryopreserved at -80 °C, and subsequently subjected to batch analysis in the central laboratory.

All patients provided signed informed consent. This study was conducted in accordance with the principles outlined in the Declaration of Helsinki and received approval from the Ethics Committee of the Affiliated Wuxi People’s Hospital of Nanjing Medical University (ethical approval no. kyl2016001).

### 2.2 Interventions and follow-up

Risk stratification and therapeutic regimen options were based on the KDIGO guideline. Patients in the RTX group received intravenous RTX (1,000 mg) on days 1 and 15. If complete remission (CR) was not achieved or CD19+ B cell counts exceeded 5 cells/mL after 6 months, an additional treatment with RTX (375 mg/m^2^) was administered. Patients who remained no remission at M6 and tested positive for ARA were switched to obinutuzumab treatment (1,000 mg on days 1 and 15, respectively). If complete remission (CR) was not achieved or the CD19+ B cell counts exceeded 5 cells/mL after 6 months, an additional treatment with obinutuzumab (375 mg/m^2^) was administered. The cyclophosphamide (CTX) group was administered with intravenous CTX (0.8–1.0 g/month) combined with oral prednisone (0.6–0.8 mg/kg/day adjusted for clinical response). All patients were treated with the maximum tolerated dose of angiotensin-converting enzyme inhibitors or angiotensin receptor blockers and followed up regularly. At M6 and M12, serum specimens were obtained from the patients before the next treatment to detect the PLA2R epitope-specific antibody (PLA2R-IgG, PLA2R-IgG4, PLA2R-CysR-IgG/IgG4, PLA2R-CTLD1-IgG/IgG4, and PLA2R-CTLD678-IgG/IgG4) anti-rituximab antibody and understand the epitope spreading.

### 2.3 Detection of PLA2R and its domain antibodies

Purified PLA2R and domain-specific proteins (CysR, CTLD1, and CTLD678) were coated onto 96-well plates (2 μg/mL, 100 μL/well) and incubated overnight at 4 °C. The next day, the plates were washed once with wash buffer, then 150 μL blocking buffer was added to each well, and the plates were blocked at room temperature for 2 h. After blocking, the plates were dried and stored at -20 °C.

Serum samples were diluted to 1:100 for -IgG antibodies and 1:20 for -IgG4 antibodies, respectively, in an assay buffer and then added to the antigen-coated plates in quadruplicate and incubated on a shaker at 25 °C for 1 h. After three washes with wash buffer, Eu^3+^-labeled goat anti-human IgG antibodies (diluted 1:100 in assay buffer) and mouse anti-human IgG4 antibodies (1:100 dilution) were added to the plates (100 μL/well) and administered according to previous protocols. After six washes, 200 μL of enhancement solution was added to the plates and agitated on a shaker at 25 °C for 5 min.

Fluorescence was measured using a DR6608 time-resolved fluorescence immunoassay (TRFIA) analyzer. The cutoff values for serum PLA2R-IgG and PLA2R-IgG4 levels were 14 and 200 ng/mL, respectively. For serum PLA2R-CysR/-CTLD1/-CTLD678-IgG, the cutoff values were 10.02, 16.01, and 14.38 RU/mL, respectively. Similarly, the cutoff values for serum PLA2R-CysR/-CTLD1/-CTLD678-IgG4 were 100, 50, and 100 RU/mL, respectively (11–14).

### 2.4 Detection of RTX antibody

TRFIA for RTX antibodies was performed using the bridging capture method (15). RTX was diluted to a concentration of 0.125 μg/mL in coating buffer, added to 96-well plates (100 μL/well), and incubated overnight at 4 °C. The next day, the plates were washed once with wash buffer, blocking buffer was added to each well, and the plates were blocked at room temperature for 2 h. After blocking, the plates were dried and stored at -20 °C. RTX was labeled with Eu^3+^ using a Eu^3+^ labeling kit according to the manufacturer’s instructions and diluted to the concentration of 1:400. Anti-rituximab idiotypic antibodies were diluted to standards in an assay buffer. Standards or serum samples (100 μL/well) were added to RTX-coated plates and incubated with agitation at 37 °C for 60 min. After two washes, 100 μL of diluted Eu^3+^-labeled RTX solution was added to each well. Subsequently, the plates were incubated with agitation at 37 °C for 60 min again and washed six times. Furthermore, 100 μL of enhancement solution was added per well, and the plates were shaken at 25 °C for 5 min. Finally, fluorescence was measured using an HG-1000 TRFIA analyzer. The cutoff value for serum ARAs was 2 ng/mL (15).

### 2.5 Definition

Remission criteria: (1) partial remission (PR) characterized by proteinuria reduction ≥50% from baseline with 24-h urinary protein <3.5 g, accompanied by stable renal function (serum creatinine fluctuation <20%); (2) CR required achievement of 24-h urinary protein <0.3 g with a normal glomerular filtration rate (GFR ≥90 mL/min/1.73 m²) or stabilization of pre-existing GFR (variation <10%); (3) non-response (NR) was defined as failure to achieve ≥50% proteinuria reduction from baseline or deterioration in renal function (serum creatinine increase >20% from baseline); and (4) immune remission was defined as PLA2R-IgG <2 RU/mL (ELISA).

Epitope spreading was defined as positive for PLA2R-CysR-IgG4 and positive for either PLA2R-CTLD1-IgG4 or PLA2R-CTLD678-IgG4 (5).

### 2.6 Statistical analyses

Statistical analyses were performed using SPSS version 27.0. To minimize data variability, PLA2R and domain-specific antibodies were natural-log-transformed. In descriptive statistics, the variables were assessed for normality using Shapiro–Wilk test. Normally distributed data are expressed as mean ± standard deviation (SD), non-normally distributed variables as median (interquartile range), and categorical variables as frequencies with percentages (*n* [%]). Qualitative variables were compared using chi-square test or Fisher’s exact test, whereas quantitative variables were compared using *t*-test or Wilcoxon–Mann–Whitney test. Chi-squared test or one-way analysis of variance was used to assess inter-sample differences. Pearson or Spearman correlation coefficient was used to evaluate correlations. Logistic regression analysis was performed to identify potential risk or protective factors for treatment response. The validity of the model was evaluated using receiver operating characteristic (ROC) curve analysis, with the area under the curve (AUC) as the primary discriminative metric. All probabilities were two-sided, and statistical significance was set at *P* < 0.05. These *P*-values were corrected for multiple comparisons by Benjamini–Hochberg to control the false discovery rate (FDR) at the 10% level.

## 3 Results

### 3.1 Characteristics of patients at baseline (M0)

A total of 74 patients with PMN diagnosed by renal biopsy were enrolled in this study. Compared with the RTX group, the CTX group appeared to have lower serum albumin (26.13 ± 6.13 vs. 21.75 ± 3.45, *P* = 0.001) and higher 24-h urinary protein levels [3.50 (2.03, 4.70) vs. 4.24 (3.88, 4.97), *P* = 0.008)]. Furthermore, PLA2R-IgG/-IgG4 and some PLA2R domain-specific antibodies showed statistically significant differences between the two groups (**Table 1**).

**Table 1:** Characteristics of PMN patients at baseline (M0).

Characteristics	Rituximab (*n* = 29)	Cyclophosphamide (*n* = 45)	*t*/*Z*/*χ* value	*P*-value
Age (years)	48.00 ± 13.83	53.36 ± 12.10	-1.757	0.083
Male (%)	17 (58.62%)	28 (62.22%)	0.096	0.757
Hypertension (%)	13 (44.83%)	24 (53.33%)	0.510	0.475
Diabetes (%)	5 (17.24%)	5 (11.11%)	0.567	0.451
Systolic BP (mmHg)	130.52 ± 18.60	132.24 ± 17.72	-0.401	0.689
Diastolic BP (mmHg)	81.38 ± 11.18	81.89 ± 10.87	-0.195	0.846
Serum albumin (g/L)	26.13 ± 6.13	21.75 ± 3.45	3.508	0.001^**^
24-h urinary protein (g/24 h)	3.50 (2.03, 4.70)	4.24 (3.88, 4.97)	-2.658	0.008^**^
Serum creatinine (μmol/L)	80.80 (62.15, 96.45)	78.00 (63.05, 86.15)	-0.775	0.438
eGFR (mL/min/1.73 m^2^)	90.37 ± 24.16	90.94 ± 20.26	-0.109	0.914
Serum uric acid (mmol/L)	353.93 ± 91.61	350.76 ± 87.62	0.149	0.882
Serum triglyceride (mmol/L)	2.10 (1.30, 3.63)	2.32 (1.70, 3.66)	-1.423	0.155
Serum cholesterol (mmol/L)	6.17 (5.45, 7.89)	7.00 (5.98, 9.27)	-1.262	0.207
Serum Low-density lipoprotein (mmol/L)	3.58(2.98, 4.30)	3.94 (2.78, 5.30)	-0.692	0.489
Hemoglobin (g/L)	127.59 ± 16.09	128.67 ± 18.40	-0.259	0.797
PLA2R-IgG^a^ (ELISA)	3.59 (2.02, 4.34)	4.76 (3.35, 5.70)	-3.480	<0.001^**^
PLA2R-IgG^a^ (TRFIA)	2.45 (0.17, 4.58)	4.92 (3.12, 5.88)	-4.091	<0.001^**^
PLA2R-IgG4^a^ (TRFIA)	6.24 (3.79, 7.86)	8.00 (5.25, 8.76)	-2.729	0.006^**^
PLA2R-CysR-IgG^a^ (TRFIA)	2.65 (0.41, 3.87)	4.17 (2.85, 5.09)	-4.003	<0.001^**^
PLA2R-CysR-IgG4^a^ (TRFIA)	5.88 ± 2.84	6.90 ± 2.25	-1.723	0.089
PLA2R-CTLD1-IgG^a^ (TRFIA)	0.03 (-1.24, 1.51)	3.65 (1.33, 4.38)	-3.997	<0.001^**^
PLA2R-CTLD1-IgG4^a^ (TRFIA)	1.53 ± 2.82	3.72 ± 2.41	-3.554	<0.001^**^
PLA2R-CTLD678-IgG^a^ (TRFIA)	0.91 ± 2.27	3.88 ± 1.21	-6.189	<0.001^**^
PLA2R-CTLD678-IgG4^a^ (TRFIA)	4.30 (-0.69, 6.15)	7.21 (6.05, 7.97)	-4.236	<0.001^**^
Epitope spreading^b^ (%)	16 (55.2%)	34 (75.6%)	3.344	0.067

Values are expressed as mean ± SD or *n* (%).

a Values that are progressed by ln.

b Definition according to -IgG4 antibodies (TRFIA).

**P < 0.01.

### 3.2 Characteristics of patients at M6 and M12 of treatment

Comparing the RTX and CTX groups, although a discrepancy in serum albumin level still existed (35.06 ± 7.33 vs. 29.49 ± 6.03, *P* < 0.001) at M6, the 24-h urinary protein levels showed no significant statistical difference at M6 (*P* = 0.379) and M12 (*P* = 0.714) (**Tables 2**, **3**) Both the CTX and RTX groups showed a decreasing trend in proteinuria over the 12-month treatment period, while serum albumin levels exhibited an increasing trend (**Supplementary Figure S1**).

**Table 2:** Characteristics of patients at M6.

Characteristics	Rituximab (*n* = 29)	Cyclophosphamide (*n* = 45)	*t*/*Z*/*χ* value	*P*-value
Serum albumin (g/L)	35.32 ± 7.34	29.49 ± 6.03	3.729	<0.001^**^
24-h urinary protein (g/24 h)	2.48 (1.73, 3.37)	1.72 (0.69, 3.51)	-0.659	0.510
Serum creatinine (μmol/L)	77.20 (60.20, 92.30)	69.20 (59.05, 82.55)	-1.727	0.084
eGFR (mL/min·1.73 m^2^)	95.60 (78.58, 109.73)	97.60 (83.35, 108.70)	-0.346	0.729
PLA2R-IgG^a^ (ELISA)	0.71 (0.32, 1.50)	1.47 (0.49, 2.91)	-1.626	0.104
PLA2R-IgG^a^ (TRFIA)	0.27 (-0.33, 1.65)	2.14 (1.57, 4.38)	-4.593	<0.001^**^
PLA2R-IgG4^a^ (TRFIA)	3.55 (3.13, 4.73)	4.05 (2.67, 8.31)	-1.220	0.222
PLA2R-CysR-IgG^a^ (TRFIA)	-0.37 ± 2.93	2.78 ± 1.65	-4.923	<0.001^**^
PLA2R-CysR-IgG4^a^ (TRFIA)	3.24 (2.03, 5.52)	3.49 (2.55, 7.05)	-1.656	0.098
PLA2R-CTLD1-IgG^a^ (TRFIA)	0.01 (-1.26, 0.45)	-0.69 (-0.69, 3.24)	-0.811	0.417
PLA2R-CTLD1-IgG4^a^ (TRFIA)	0.54 ± 2.20	2.54 ± 2.61	-3.104	0.003^**^
PLA2R-CTLD678-IgG^a^ (TRFIA)	-1.27 (-3.91, -0.24)	2.06 (1.55, 2.81)	-5.684	<0.001^**^
PLA2R-CTLD678-IgG4^a^ (TRFIA)	0.00 (-0.51, 2.36)	3.40 (2.34, 6.02)	-3.368	<0.001^**^
Epitope spreading^b^ (%)	3 (10.34%)	11 (24.44%)	6.026	0.014^*^
Proteinuria remission (%)	11 (37.93%)	27 (60.00%)	3.438	0.064
Immunological remission^c^ (%)	25 (86.21%)	29 (74.36%)	1.428	0.232
ΔPLA2R-IgG (TRFIA)	0.93 (0.65, 0.97)	0.63 (0.47, 0.87)	-2.019	0.043^*^
ΔPLA2R-IgG4 (TRFIA)	0.90 (0.33, 0.97)	0.92 (0.51,0.99)	-0.836	0.403
ΔPLA2R-CysR-IgG (TRFIA)	0.94 (0.72, 0.98)	0.58 (0.34, 0.87)	-3.118	0.002^**^
ΔPLA2R-CysR-IgG4 (TRFIA)	0.91 (0.30, 0.98)	0.80 (0.40, 0.92)	-0.956	0.339
ΔPLA2R-CTLD1-IgG (TRFIA)	0.20 (-0.47, 0.85)	0.10 (0.00, 0.97)	-1.104	0.270
ΔPLA2R-CTLD1-IgG4 (TRFIA)	0.67 (-1.44, 0.96)	0.69 (-0.48, 0.96)	-0.054	0.957
ΔPLA2R-CTLD678-IgG (TRFIA)	0.96 (0.62, 0.99)	0.78 (0.61, 0.92)	-2.107	0.035^*^
ΔPLA2R-CTLD678-IgG4 (TRFIA)	0.91 (-1.19, 1.00)	0.98 (0.72, 0.99)	-0.428	0.669

Values are expressed as mean ± SD or *n* (%). Δ(%) = (M0- M6)/M0 * 100%.

a Values that are progressed by ln.

b Definition according to -IgG4 antibodies (TRFIA).

c Serum PLA2R-IgG was detected by ELISA.

*P < 0.05; **P < 0.01.

**Table 3:** Characteristics of patients at M12.

Characteristics	Rituximab (*n* = 25)	Cyclophosphamide (*n* = 45)	*t*/*Z*/*χ* value	*P*-value
Serum albumin (g/L)	39.86 ± 5.43	35.19 ± 7.72	2.965	0.004^**^
24-h urinary protein (g/24 h)	0.99 (0.33, 2.24)	0.87 (0.13, 2.10)	-0.367	0.650
Serum creatinine (μmol/L)	73.40 (61.30,90.83)	72.40 (60.75,78.60)	-0.901	0.368
eGFR (mL/min/1.73 m^2^)	99.50 (74.18,110.10)	97.90 (84.10,109.40)	-0.148	0.882
Proteinuria remission (%)	16 (61.5%)	31 (75.6%)	1.504	0.220
Immunological remission^a^ (%)	21 (96.2%)	22 (65.6%)	5.732	0.017^*^

Values are expressed as mean ± SD or *n* (%).

a Serum PLA2R-IgG was detected by ELISA.

*P < 0.05; **P < 0.01.

Moreover, PLA2R-IgG (*P* < 0.001), PLA2R-CysR-IgG (*P* < 0.001), PLA2R-CTLD678-IgG (*P* < 0.001), and PLA2R-CTLD678-IgG4 (*P* < 0.001) showed lower levels in the RTX group, which had fewer patients with epitope spreading (20.7% vs. 26.7%, *P* < 0.001) at M6. Although no statistical differences in proteinuria remission rates were found at M6 (37.9% vs. 60.0%, *P* = 0.875) and M12 (61.5% vs. 75.6%, *P* = 0.220) in the two groups, the immunological remission rates (96.2% vs. 65.6%, *P* = 0.017) defined by PLA2R-IgG (ELISA) at M12 was higher in the RTX group. Simultaneously, as shown in the scatter plot and the table, the clearance rates from M0 to M6 of antibodies, including PLA2R-IgG (*P* = 0.043), PLA2R-CysR-IgG (*P* = 0.002), and PLA2R-CTLD678-IgG (*P* = 0.035) were also higher in the RTX group (**Tables 2**, **3**; **Supplementary Figures S2**, **S3**).

### 3.3 Analysis of remission in the RTX group

In the RTX group, the serum albumin levels (*P* < 0.001) decreased significantly at M6, whereas the level of proteinuria showed no statistical difference. Compared with the data at baseline, PLA2R-IgG (ELISA) (*P* <0.001), PLA2R-IgG (TRFIA) (*P* = 0.003), PLA2R-IgG4 (*P* = 0.001), PLA2R-CysR-IgG (*P*<0.001), PLA2R-CysR-IgG4 (*P* = 0.001), PLA2R-CTLD678-IgG (*P* < 0.001), and PLA2R-CTLD678-IgG4 (*P* = 0.031) significantly declined at 6 months, and epitope spreading also appeared to be significantly reduced (*P* = 0.027). After 6 months of RTX therapy, ARAs were detected in four patients (**Table 4**).

**Table 4:** Characteristics of the RTX group (M0 vs. M6).

Characteristics	M0 (*n* = 29)	M6 (*n* = 29)	*t*/*Z*/*χ* value	*P*-value
Serum albumin (g/L)	26.13 ± 6.13	35.32 ± 7.34	-5.181	<0.001^**^
24-h urinary protein (g/24 h)	3.63 ± 1.88	2.38 ± 1.44	2.842	0.006^**^
Serum creatinine (μmol/L)	80.80 (62.15, 96.45)	77.20 (60.20, 92.30)	-0.225	0.822
eGFR (mL/min·1.73 m^2^)	95.10 (69.45, 108.35)	95.60 (78.58, 109.73)	-0.04	0.968
PLA2R-IgG^a^ (ELISA)	3.59 (2.02, 4.34)	0.71 (0.32, 1.50)	-4.153	<0.001^**^
PLA2R-IgG^a^ (TRFIA)	2.45 (0.17, 4.58)	0.27 (-0.33, 1.65)	-3.009	0.003^**^
PLA2R-IgG4^a^ (TRFIA)	6.24 (3.79, 7.86)	3.55 (3.13, 4.73)	-3.196	0.001^**^
PLA2R-CysR-IgG^a^ (TRFIA)	2.65 (0.41, 3.87)	-0.37 (-3.00, 1.41)	-3.387	<0.001^**^
PLA2R-CysR-IgG4^a^ (TRFIA)	5.88 ± 2.84	3.56 ± 2.22	3.457	0.001^**^
PLA2R-CTLD1-IgG^a^ (TRFIA)	0.25 ± 2.21	-0.31 ± 1.49	1.121	0.267
PLA2R-CTLD1-IgG4^a^ (TRFIA)	1.53 ± 2.82	0.54 ± 2.20	1.472	0.147
PLA2R-CTLD678-IgG^a^ (TRFIA)	0.91 (-0.80, 2.55)	-1.27 (-3.91, -0.24)	-3.802	<0.001^**^
PLA2R-CTLD678-IgG4^a^ (TRFIA)	4.30 (-0.69, 6.15)	0 (-0.51, 2.36)	-2.163	0.031^*^
Epitope spreading^b^ (%)	15 (51.7%)	3 (10.3%)	11.600	<0.001^**^
ARA positive rate (%)	0	4 (13.8%)	5.100	0.024^*^

Values are expressed as mean ± SD or *n* (%).

a Values that are progressed by ln.

b Definition according to -IgG4 antibodies (TRFIA).

*P < 0.05; **P < 0.01.

A further comparison between the proteinuria remission and non-remission groups after 6 months of RTX treatment revealed that PLA2R-IgG (TRFIA) (*P* = 0.049) and PLA2R-CysR-IgG4 (*P* = 0.018) were lower in the CR/PR group. Notably, epitope spreading was eliminated in the CR/PR group while remaining in the NR group (0% vs. 14.3%). Four ARA-positive PMN patients (0% vs. 22.2%, *P* = 0.009) were identified in the NR group by detecting ARAs at M6 (**Table 5**; **Supplementary Figure S4**).

**Table 5:** Characteristics of the RTX group at baseline (M6).

Characteristics	Complete or partial remission (*n* = 11)	No remission (*n* = 18)	*t*/*Z*/*χ* value	*P*-value
Serum albumin (g/L)	39.15 ± 5.34	32.99 ± 7.53	2.366	0.025*
24-h urinary protein (g/24 h)	0.48 (0.05, 2.84)	3.09 (2.27, 3.76)	0.015	0.014^*^
Serum creatinine (μmol/L)	65.10 (55.70, 90.50)	78.75 (70.43, 95.85)	0.126	0.134
eGFR (mL/min/1.73 m^2^)	101.55 ± 16.62	84.40 ± 20.01	1.763	0.090
PLA2R-IgG^a^ (ELISA)	0.38 (0.28, 1.27)	0.79 (0.43, 2.58)	0.072	0.076
PLA2R-IgG^a^ (TRFIA)	0.06 (-0.45, 0.27)	1.04 (-0.23, 2.03)	0.048	0.049^*^
PLA2R-IgG4^a^ (TRFIA)	3.29 (3.11, 3.64)	3.93(3.22,5.27)	0.088	0.092^*^
PLA2R-CysR-IgG^a^ (TRFIA)	-1.74 ± 2.21	0.43 ± 3.05	-1.962	0.061
PLA2R-CysR-IgG4^a^ (TRFIA)	2.35 ± 1.60	4.31 ± 2.26	-2.513	0.018^*^
PLA2R-CTLD1-IgG^a^ (TRFIA)	-0.70 ± 1.43	-0.07 ± 1.52	-1.104	0.279
PLA2R-CTLD1-IgG4^a^ (TRFIA)	-1.41 (-2.30, 1.02)	1.15 (-0.06, 2.24)	0.061	0.064
PLA2R-CTLD678-IgG^a^ (TRFIA)	-2.27 ± 1.78	-1.75 ± 2.48	-0.917	0.368
PLA2R-CTLD678-IgG4^a^ (TRFIA)	0.00 (-0.36, 1.93)	0.05 (-0.56, 2.84)	0.857	0.877
Epitope spreading^b^ (%)	0 (0.0%)	3 (16.67%)	2.045	0.153
ARA positive rate (%)	0 (0.0%)	4 (22.2%)	5.333	0.021^*^

Values are expressed as mean ± SD or *n* (%).

a Values that are progressed by ln.

b Definition according to -IgG4 antibodies (TRFIA).

*P < 0.05.

### 3.4 Correlation analysis of the total PLA2R-IgG/IgG4 and the domain-specific antibodies of PLA2R

The correlation analysis revealed that the total PLA2R-IgG/IgG4 was significantly associated with PLA2R-CysR-IgG/IgG4 and PLA2R-CTLD678-IgG. Spearman’s correlations of PLA2R-IgG with PLA2R-CysR-IgG/IgG4 and PLA2R-CTLD-IgG were 0.703, 0.730, and 0.522, respectively, and the correlations of the PLA2R-IgG4 with the same antibodies mentioned above were 0.723, 0.792, and 0.545, respectively (**Supplementary Figure S5**).

### 3.5 Analysis of factors related to proteinuria remission

We explored 25 clinical factors associated with proteinuria remission, including serum albumin, PLA2R-IgG/-IgG4, its domain-specific antibodies, and epitope spreading. In the univariate logistic regression analyses, it was found that eGFR (*P* = 0.045), PLA2R-IgG (ELISA) (*P* = 0.025), PLA2R-IgG (TRFIA) (*P* = 0.036), PLA2R-IgG4 (TRFIA) (*P* = 0.033), PLA2R-CysR-IgG (TRFIA) (*P* = 0.017), and epitope spreading (*P* = 0.004) were correlated with proteinuria remission statistically, and after Benjamini–Hochberg correction, epitope spreading remained. Multivariable logistic regression analysis of the abovementioned relevant variables revealed that epitope spreading (*P* = 0.031) was an independent risk factor for proteinuria remission at M6 after adjusting for baseline eGFR and PLA2R concentrations (**Tables 6**, **7**). The ROC curve of the regression equation demonstrated an AUC of 0.932 (**Supplementary Figures S5**, **S6**; **Table 8**).

**Table 6:** Univariate logistic regression analyses of proteinuria remission factors.

Characteristics	*β*	OR (95% CI)	*P*
Epitope spreading^a^	2.757	15.750 (2.373, 104.537)	0.004^**^
PLA2R-IgG (ELISA)	-0.036	0.965 (0.935, 0.995)	0.025^*^
PLA2R-IgG (TRFIA)	-0.034	0.967 (0.937, 0.998)	0.036^*^
PLA2R-IgG4 (TRFIA)	-0.001	0.999 (0.999, 1.000)	0.033^**^
PLA2R-CysR-IgG (TRFIA)	-0.066	0.936 (0.886, 0.988)	0.017^*^
PLA2R-CTLD1-IgG4 (TRFIA)	-0.125	0.883 (0.734, 1.062)	0.187^*^
eGFR	0.045	1.046 (1.001, 1.093)	0.045^*^

a Definition according to PLA2R-IgG4 antibodies (TRFIA).

*P < 0.05; **P < 0.01.

**Table 7:** Multivariate logistic regression analysis of proteinuria remission factors.

Characteristics	*β*	OR (95% CI)	*P*
Epitope spreading^a^	3.140	23.109 (1.342, 397.909)	0.031^*^
PLA2R-IgG(ELISA)	-0.051	0.951 (0.909, 0.994)	0.027^*^
eGFR	0.071	1.074 (1.000, 1.153)	0.051
Constant	-7.259	0.001	0.049^*^

a Definition according to PLA2R-IgG4 antibodies (TRFIA).

*P < 0.05.

**Table 8:** ROC curve analysis of multivariate logistic regression analysis.

AUC	*P*-value	95% CI	Optimal cutoff value	Sensitivity	Specificity
0.932	0.001^**^	(0.841, 1.000)	0.833	1.000	0.833

**P < 0.01.

### 3.6 Outcome of the PMN patients who were ARA-positive

In this study, ARAs were detected in four patients after 6 months of RTX therapy, and none of them had achieved CR/PR at M6. Based on the clinical condition and wishes of the patients, replacement of treatment with obinutuzumab was put into effect. All patients achieved CR or PR within 3–9 months. Although the follow-up period was short, one of these patients (case 1) achieved CR at M9 after receiving 2,000 mg of obinutuzumab (**Table 9**).

**Table 9:** Characteristics of the patients using obinutuzumab.

Case	Gender	Age,year	Rituximab cumulative dose, mg	Anti-rituximab-IgG, ng/mL	Obinutuzumab dose, mg	24-h urinary protein (g/24 h)				Outcome
						M0^a^	M3	M6	M9	
1	Male	58	2,600	87.6	3,000	3.95	1.86	1.74	0.29	CR
2	Female	56	2,000	122.99	2,000	7.73	1.43	2.30	/	PR
3	Male	58	3,000	5.88	2,000	6.24	2.22	1.66	/	PR
4	Male	68	2.000	2.44	2,000	6.21	2.06	1.27	/	PR

a Before the treatment with obinutuzumab.

## 4 Discussion

PMN is induced by antigen–antibody binding and deposition in the basilar membrane. Targeted treatment regimens have emerged with the discovery of pathogenic mechanisms. The 2021 KIDGO guidelines recommend that patients assessed as moderate and high risk according to risk stratification use RTX as the first-line treatment, whereas CTX is used in very-high-risk patients. The efficacy of RTX has also been confirmed (16–19). However, some patients using RTX still do not achieve CR or PR. Therefore, this study aimed to identify the best prognostic predictor in patients with PMN treated with RTX, which will contribute to the establishment of a clinical prediction model.

In the study, there was no statistical difference in the clinical remission between the CTX and RTX groups either at M6 (37.9% vs. 60.0%, *P* = 0.875) or M12 (75.6% vs. 61.5%, *P* = 0.220), while for immunological remission, the RTX group demonstrated a higher immunological remission rate at M12 (96.2% vs. 65.6%, *P* = 0.017) and more efficient PLA2R antibody clearance rate at M6, which may be due to the direct effect of RTX targeting CD20+ B cells. Proteinuria remission occurs later than immunological remission in PMN patients (6, 20, 21), suggesting that further validation of the efficacy of RTX treatment by extending the follow-up observation period is of great concern.

In a previous study, the clinical remission rates at M12 reported in the cohort of GMERITUX (17), MENTOR (19), and RI-CYCLO (18) were 64.9%, 60%, and 62%, respectively. In a prospective study (22), 43 of 70 patients (61.43%) in the RTX group and 54 of 71 patients (76.06%) in the CTX group achieved CR/PR (*P* = 0.06) at M12, with similar remission rates at M24 (75.41% vs. 68.97%, *P* = 0.54). In another study (23), compared with those in the NR group, PLA2R-IgG and PLA2R-CysR-IgG titers declined more in the PR or CR groups at M6 and M12, respectively. These results are consistent with the conclusions of the present study.

Despite differences in baseline characteristics between the RTX and CTX groups, given the aforementioned inconsistency between clinical and immunological remission, we decided to conduct a further analysis of the RTX group. In the subsequent analysis, compared with the RTX group at M0, it showed lower titers of PLA2R-specific antibodies and a lower positive rate of epitope spreading at M6, which may be related to the efficacy of RTX. Next, the RTX group was divided into CR/PR and NR groups based on the proteinuria remission of M6, which displayed PLA2R-IgG (*P* = 0.049) and PLA2R-CysR-IgG4 (*P* = 0.018) titers; the positive rate of epitope spreading (16.67%) was significantly higher in the NR group.

To further explore the relationship of PLA2R-specific antibodies and epitope spreading with proteinuria remission, we performed univariate logistic analysis and found that six indicators, including PLA2R-IgG (ELISA), PLA2R-IgG/-IgG4 (TRFIA), PLA2R-Cys-IgG, PLA2R-CTLD1-IgG4, and epitope spreading, were associated with clinical remission, and epitope spreading was still retained after Benjamini–Hochberg correction. Further multivariate logistic regression analysis (AUC-ROC = 0.932) showed that epitope spreading was an independent risk factor for proteinuria remission at M6. Experimental evidence that epitope spreading may occur and influence the severity of MN was documented in a Heymann nephritis model of MN in rats, which coincided with worsening proteinuria (24). Simultaneously, as an autoimmune disease, a proportion of PLA2R-MN cases show spreading of the immunodominant epitope from -CysR to -CTLD1 and/or -CTLD678, which is related to a worse prognosis (25).

In the NICE cohort (16), lower epitope spreading at diagnosis was associated with proteinuria remission at M6 (50% vs. 76%, *P* = 0.05), whereas epitope spreading at M0 (100% vs. 48%, *P* = 0.01) and incomplete clearance of PLA2R-IgG at M6 (0% vs. 9%, *P* = 0.05) were associated with recurrence. Another study conducted by Seitz-Polski et al. (25) reported that epitope spreading beyond the CysR epitope was an independent risk factor for poor renal prognosis. In a recent prospective study, the multi-domain recognition exhibited a reduced remission rate compared with the single-domain (44.44% vs. 82.61%, *P* = 0.011). Subsequent to the Kaplan–Meier analysis, it was demonstrated that the multi-domain group exhibited lower remission rates in comparison to the single-domain group at a number of designated time points (26). In contrast, Reinhard considered that the levels of domain-specific PLA2R antibodies against -CysR, -CTLD1, and -CTLD7 were strongly correlated with the levels of total anti-PLA2R1 antibodies but could not predict prognosis independtly of the total anti-PLA2R1 antibody levels (6). Similarly, in an Italian study, Ruggenenti et al. found that lower PLA2R-IgG (*P* = 0.002) and PLA2R-CysR-IgG (*P* = 0.001) titers at M0 were associated with a higher probability of clinical remission, whereas PLA2R-CTLD-IgG was not associated with remission (23). Our study confirmed that epitope spreading was an independent risk factor for proteinuria remission.

Although RTX could induce seasonable immunological remission, some patients did not maintain remission. In this study, four ARA antibody-positive cases were detected in the NR group after 6 months of treatment with RTX. Based on the wishes of the four patients, we changed the treatment regimen to obinutuzumab, and all four patients achieved PR or CR in 3 to 9 months of follow-up. As a fully human anti-CD20 monoclonal antibody, obinutuzumab is more likely to avoid the effects of anti-rituximab antibodies, depending on the immune response profiles of the different organisms.

In a clinical combined with *in vitro* experimental study, ARA was detected in 10 of 44 RTX-treated patients with PMN by ELISA, and in an *in vitro* test against ARA, anti-CD20 monoclonal antibody efficacy including obinutuzumab, ocrelizumab, and ofatumumab was not impaired in eight patients, while three ARA-positive patients were successfully treated with ofatumumab (9). To explore the factors influencing ARA production, Maxime Teisseyre et al. detected ARA in 21 (58%) out of 55 patients included and found that the patients in the ARA-positive group had a higher epitope spreading rate (82% vs. 44%, *P* < 0.05) (27). Epitope spreading indicates that the organism of that patient is more immunologically active, which means that it is more likely to produce ARA, and ARA-positive patients are more likely to experience failure of RTX treatment, consistent with the findings of the present study as previously described.

Xiaofan Hu et al. conducted a study that compared the efficacy of RTX and obinutuzumab. In a subgroup analysis of PLA2R-associated PMN, patients in the obinutuzumab group were more likely to reach immunological remission at M6 (92% vs. 64%, *P* = 0.06) (28). In a retrospective study in China (29), the response rates were significantly higher in 51 patients treated with obinutuzumab than in those treated with RTX (90.0% vs. 38.7%, *P* < 0.001) over a 24-month follow-up period. Cox proportional hazards survival regression analysis also showed superior efficacy of obinutuzumab (*P* < 0.001). All of these studies have shown that the efficacy of obinutuzumab is superior to that of RTX, but the choice of obinutuzumab treatment was not based on a positive ARA test but rather due to failure or resistance to treatments such as glucocorticoids, which is known as refractory MN. In contrast, all four patients in the present study selected obinutuzumab treatment because of positive ARA test results. Therefore, biomarkers or clinical indicators, such as ARA and epitope spreading, that can be used to determine the timing of selection of obinutuzumab or other fully human anti-CD20 monoclonal antibodies need to be explored further.

The limitations of this study include its retrospective design and a small number of patients in the RTX group, and some patients had a short follow-up observation period, which means that the conclusions of this study need to be confirmed by a larger sample size and long-term clinical studies.

## 5 Conclusion

In conclusion, compared with CTX, RTX induced a higher rate of immunological remission at M12. Epitope spreading of PLA2R was identified as an independent risk factor for clinical remission after 6 months of treatment with RTX.

## Data Availability

The raw data supporting the conclusions of this article will be made available by the authors, without undue reservation.
