# Supplementary material for: Clinical study of PLA2R epitope spreading for predicting proteinuria remission in primary membranous nephropathy
Source: Front Immunol. 2025 Oct 22;16:1685738. doi: 10.3389/fimmu.2025.1685738 (PMC12586004; doi:10.3389/fimmu.2025.1685738)
Supplement: Supplementary file 1 [file Image1.pdf]

Supplementary Material

1 Supplementary Figures

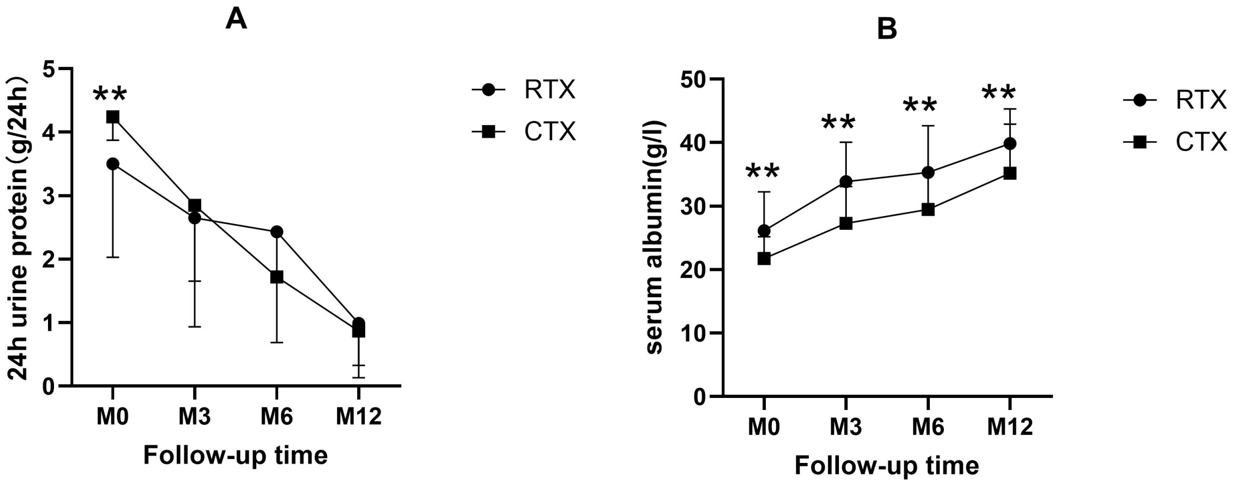

Supplementary Figure 1. Changes and comparations in 24h urinary protein(A) and serum albumin(B) levels in the RTX and CTX groups during the 12 months of follow-up.

A: 24h urinary protein in the RTX and CTX groups during 12months of follow-up. (median interquartile range, **P＜0.01)

B: Serum albumin in the RTX and CTX groups during 12months of follow-up. (mean±SD, **P＜0.01)

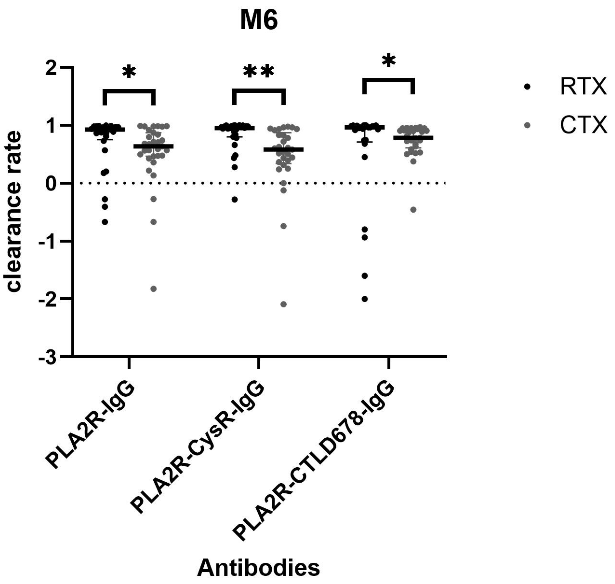

Supplementary Figure 2. The scatter plots and comparations of the clearance rates of PLA2R-IgG, PLA2R-CysR-IgG, and PLA2R-CTLD678-IgG in the RTX and CTX groups at M6. The clearance rates from M0 to M6 of PLA2R-IgG (P=0.043), PLA2R-CysR-IgG (P=0.002), and PLA2R-CTLD678-IgG (P=0.035) were higher in the RTX group. (*P<0.05, **P<0.01)

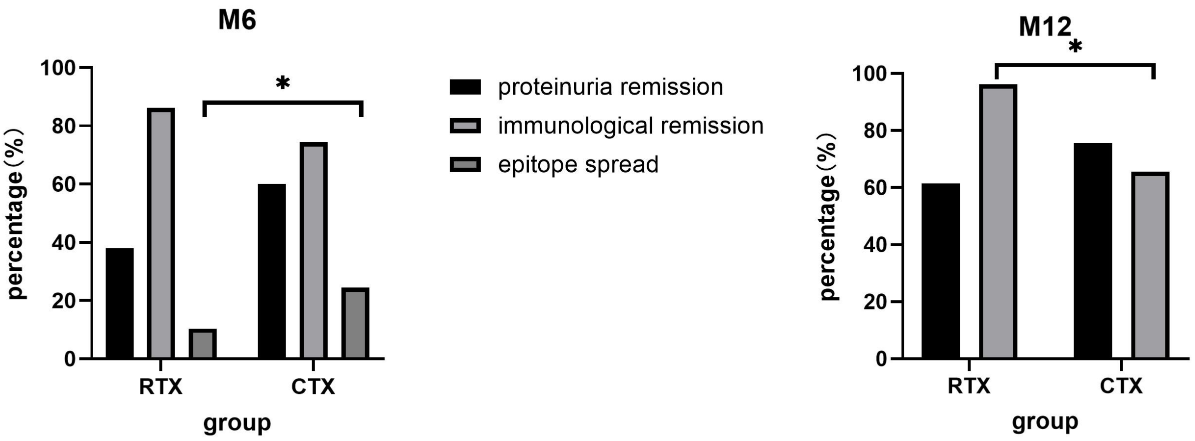

Supplementary Figure 3. Comparations between the proteinuria remission, immunological remission, and epitope spread rates of the RTX and CTX groups at M6 and M12(*P<0.05). RTX group had fewer patients with epitope spreading (20.7% vs. 26.7%, P<0.001) at M6. The immunological remission rates (96.2% vs. 65.6%, P=0.017) defined by PLA2R-IgG (ELISA) at M12 was higher in the RTX group.

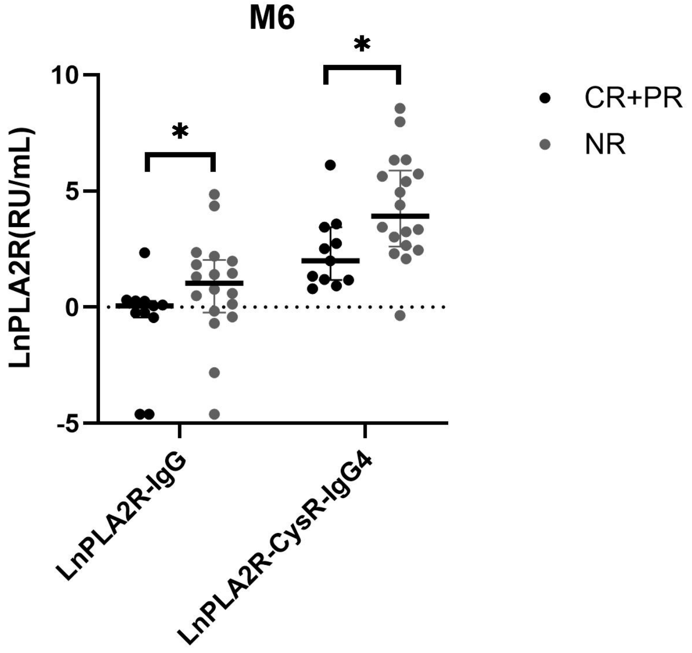

Supplementary Figure 4. The scatter plots and comparations of lnPLA2R-IgG and lnPLA2R-CysR-IgG4 in the CR+PR and NR groups of the RTX group at M6(*P＜0.05). The PLA2R-IgG (TRFIA) (P=0.049) and PLA2R-CysR-IgG4 (P=0.018) were lower in the CR/PR group.

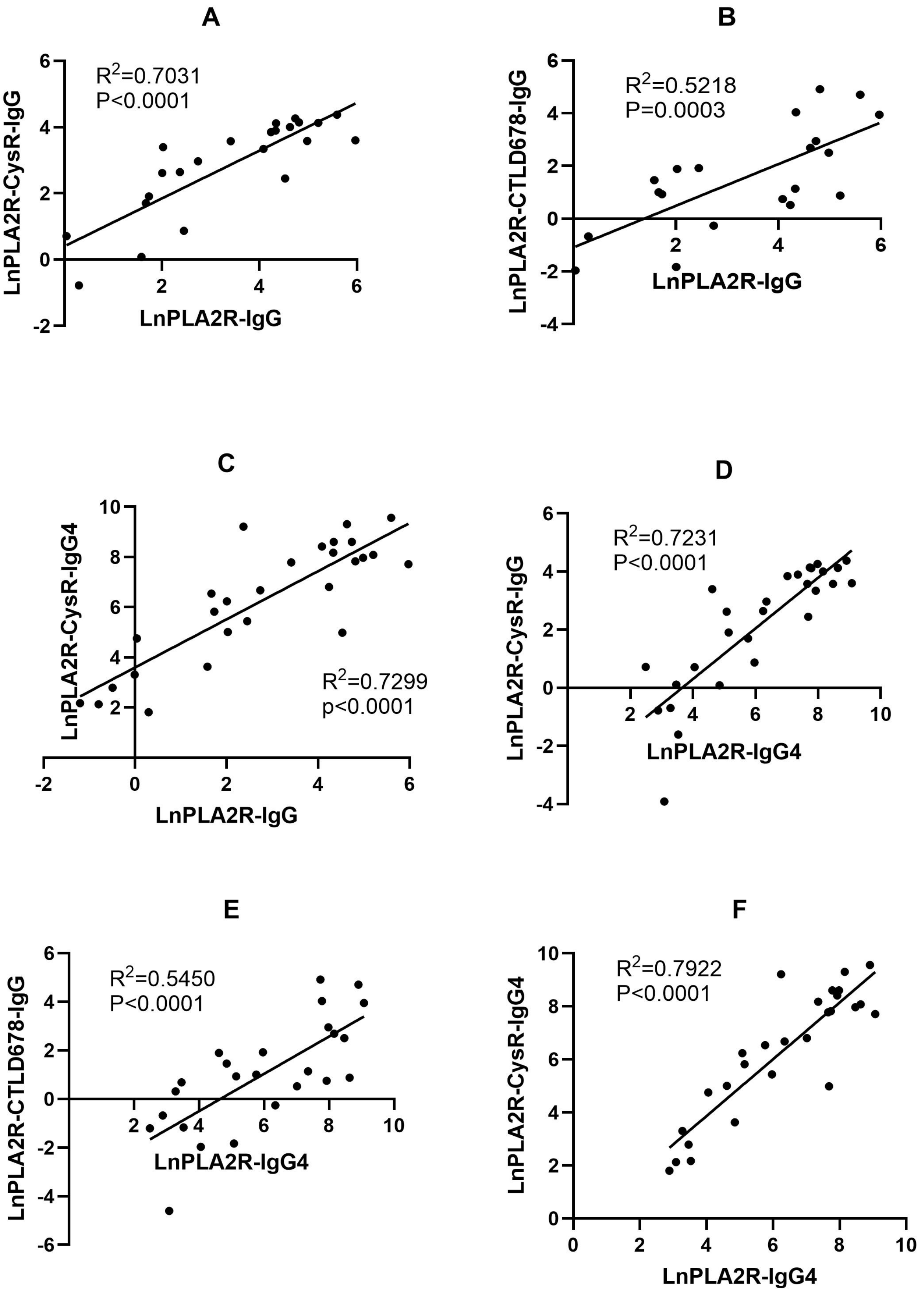

Supplementary Figure 5. The scatter plots for the correlation analyses of the PLA2R-IgG/IgG4 with

the domain-specific epitope antibodies.(Only scatter plots with at least moderate correlations are listed, that is $R^2 > 0.5$)

A: The correlation analysis between LnPLA2R-IgG and LnPLA2R-CysR-IgG at M0. ($R^2 = 0.7031$, $P < 0.0001$)

B: The correlation analysis between LnPLA2R-IgG and LnPLA2R-CTLD678-IgG at M0.($R^2 = 0.5218$, $P = 0.0003$)

C: The correlation analysis between LnPLA2R-IgG and LnPLA2R-CysR-IgG4 at M0. ($R^2 = 0.7299$, $P < 0.0001$)

D: The correlation analysis between LnPLA2R-IgG4 and LnPLA2R-CysR-IgG at M0. ($R^2 = 0.7231$, $P < 0.0001$)

E: The correlation analysis between LnPLA2R-IgG4 and LnPLA2R-CTLD678-IgG at M0. ($R^2 = 0.5450$, $P < 0.0001$)

F: The correlation analysis between LnPLA2R-IgG4 and LnPLA2R-CysR-IgG4 at M0. ($R^2 = 0.7922$, $P < 0.0001$)

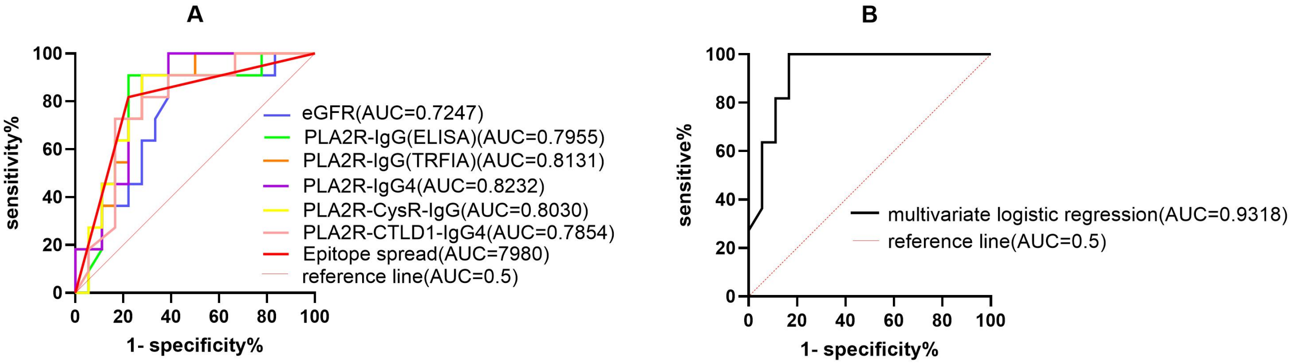

Supplementary Figure 6. The ROC curve of the logistic regression analyses.

A: The ROC curve for univariate logistic regression analyses of eGFR (AUC = 0.725), PLA2R-IgG (ELISA) (AUC = 0.796), PLA2R-IgG (TRFIA) (AUC = 0.813), PLA2R-IgG4 (TRFIA) (AUC = 0.823), PLA2R-CysR-IgG (TRFIA) (AUC = 0.803), PLA2R-CTLD1-IgG4 (TRFIA) (AUC = 0.785), and epitope spreading (AUC = 0.798).

B: The ROC curve of the multivariate logistic regression analyses including eGFR, PLA2R-IgG and epitope spreading. (AUC = 0.932)
